# YtnP lactonase from *Bacillus licheniformis* T2 mitigates virulence and biofilm formation in carbapenem-resistant *Acinetobacter baumannii* via quorum sensing quenching

**DOI:** 10.3389/fmicb.2026.1892272

**Published:** 2026-09-22

**Authors:** Bin Liu, Caihua Li, Yizhen Zhu, Mengxia Zhu, Jilai Yin, Lei Liu

**Affiliations:** 1 Key Laboratory of Emergency and Trauma of Ministry of Education, Department of Pulmonary and Critical Care Medicine, The First Affiliated Hospital, Hainan Medical University, Haikou, China; 2 Key Laboratory of Emergency and Trauma of Ministry of Education, Department of Microbiology Laboratory, The First Affiliated Hospital, Hainan Medical University, Haikou, China

**Keywords:** anti-virulence therapy, biofilm disruption, carbapenem-resistant *Acinetobacter baumannii*, quorum quenching, YtnP

## Abstract

**Backgroud:**

Carbapenem-resistant *Acinetobacter baumannii* (CRAB) has become a predominant nosocomial pathogen worldwide, with biofilm formation and quorum sensing (QS) primarily responsible for its high antimicrobial tolerance and clinical refractory infections. Targeting bacterial virulence rather than conventional bactericidal strategies represents a promising alternative to combat multidrug-resistant CRAB.

**Methods:**

In this study, we isolated a N-acyl homoserine lactonase YtnP from mangrove rhizosphere-derived *Bacillus licheniformis* T2. We comprehensively biochemically characterized the YtnP and systematically validated its potent quorum-quenching, antibiofilm and anti-virulence activities against clinical CRAB strains both *in vitro* and *in vivo* model.

**Results:**

YtnP is a 280-amino-acid metalloenzyme of approximately 30 kDa containing a conserved HXHXDH motif, exhibiting optimal activity at 37°C –45°C and pH 7.0–7.5. Distinct from classical lactonases, YtnP maintained high catalytic activity without exogenous Zn^2+^ supplementation but was markedly inhibited by EDTA and excess Zn^2+^. Functional assays revealed that YtnP efficiently degraded the key QS signal 3-OH-C12-HSL with a degradation rate of 59.5% without affecting the planktonic growth of CRAB. YtnP significantly dismantled mature CRAB biofilms, reducing biofilm biomass by 55.56% and maximum thickness by 61.76%, and prominently downregulated the transcription of virulence-related outer membrane protein genes *ompA* and *csuA/B* by nearly 50% and 52.6%, respectively. In the *Galleria mellonella* model, YtnP remarkably alleviated CRAB metabolite-induced lethality and increased the 36-h larval survival rate nearly sixfold.

**Conclusion:**

Collectively, mangrove-derived YtnP acts as a potent quorum-quenching enzyme that suppresses CRAB biofilm formation and virulence in a non-bactericidal manner. This work highlights the potential of YtnP as a promising anti-virulence adjuvant candidate for the development of alternative therapeutic strategies against intractable CRAB infections.

## Introduction

*Acinetobacter baumannii*, particularly carbapenem-resistant strains (CRAB), has emerged as a critical global nosocomial pathogen, posing a severe threat to public health due to its propensity to cause life-threatening infections, including pneumonia, bacteremia, and wound infections (Boutzoukas and Doi, 2025; Russo and Serapide, 2025). The clinical management of CRAB is exceedingly challenging, driven by its extensive multidrug resistance profiles (Asmare et al., 2025). Recent surveillance data underscore that CRAB exhibits resistance to most last-line antibiotics, leaving clinicians with severely limited therapeutic options (Qin et al., 2024). In this context, the development of novel therapeutic strategies targeting bacterial virulence mechanisms, rather than relying solely on bactericidal activity, represents a paradigm shift to combat this public health crisis (Gonzales et al., 2024; Lu et al., 2025).

Biofilm formation is a central virulence trait that underpins the persistence and chronic nature of CRAB infections (Mea et al., 2021). Within biofilms, bacterial cells are embedded in a self-produced extracellular polymeric substance (EPS) matrix that acts as a physical and chemical barrier against host immune clearance and severely limits antimicrobial penetration, generating a state of pronounced antimicrobial tolerance rather than exerting direct genetic resistance (Ciofu et al., 2022). The biofilm niche also favors horizontal gene transfer, thereby accelerating the dissemination of antibiotic-resistance determinants within poly-microbial consortia (Patil et al., 2021).

In CRAB, these processes are coordinated by the LuxI/LuxR-type quorum sensing (QS) circuit centered on the divergent *abaI/abaR* locus (Pumirat et al., 2024; Xiong et al., 2022). The *abaI*-derived autoinducer N-(3-hydroxy-dodecanoyl)-L-homoserine lactone (3-OH-C12-HSL) activates the intracellular receptor AbaR, which transcriptionally governs downstream effectors–most notably the major outer membrane adhesin OmpA and the CsuA/B chaperone–usher pilus system–that drive initial surface attachment, EPS matrix anchoring, and architectural maturation (Nie et al., 2020; Tomaras et al., 2008). Crucially, genetic disruption of *abaI* selectively impairs biofilm formation and virulence phenotypes without abolishing basal growth, validating the QS axis as a tractable “disarmable” node whose targeted quenching offers a much-needed, resistance-sparing route to undermine CRAB persistence (Rather et al., 2021; Zhong and He, 2021). Given the high regional prevalence of CRAB and the urgent demand for alternative anti-virulence approaches, we screened clinical isolates to select a strain with a functionally intact QS system for subsequent mechanistic study.

Accordingly, quorum quenching (QQ)–the enzymatic degradation or receptor antagonism of QS signaling–has emerged as a leading anti-virulence paradigm, shifting the therapeutic goal from bactericidal eradication to virulence disarmament (Djokic et al., 2022; Mayer et al., 2018). Among QQ effectors, N-acyl homoserine lactonases stand out; these enzymes catalyze irreversible hydrolysis of the lactone ring of AHL signal molecules to block quorum-sensing signal transduction (Fortaleza et al., 2026). Although well-characterized lactonase AiiA has been exploited to control aquaculture pathogens (Peng et al., 2021), prior work has verified that YtnP-type enzymes can degrade the key QS signal 3-OH-C12-HSL produced by *A. baumannii* (Mayer et al., 2018).

Multiple prior studies further reported that YtnP-family lactonases from *Bacillus* spp. are dominant functional AHL-degrading enzymes with favorable soluble recombinant expression, which greatly facilitates *in vitro* and *in vivo* functional research (Djokic et al., 2022; Mayer et al., 2018). However, most available investigations only employed standard laboratory strains rather than clinical CRAB isolates, and few provided integrated biochemical profiling together with *in vivo* virulence validation. These research gaps inspired our core hypothesis: mangrove rhizosphere-derived *B. licheniformis* T2 YtnP could effectively interfere with the AbaI/AbaR quorum-sensing cascade in clinical CRAB, thereby disrupting biofilm formation and attenuating bacterial virulence. Accordingly, we conducted systematic biochemical, phenotypic and *in vivo* experiments to comprehensively characterize the anti-virulence efficacy of this mangrove-derived YtnP against clinical CRAB isolates.

Herein, we fully characterize the biochemical properties of a N-acyl homoserine lactonase from mangrove rhizosphere-isolated *Bacillus licheniformis* T2. We further systematically verify its potent activity in quenching 3-OH-C12-HSL-dependent QS, disassembling mature CRAB biofilms, and mitigating CRAB pathogenicity in the *Galleria mellonella* larva model, filling the unaddressed research gap regarding integrated *in vitro* and *in vivo* functional assessment of mangrove-derived YtnP against clinical CRAB.

## Materials and methods

### Screening and identification of quorum quenching bacteria from environmental samples

*Bacillus licheniformis* T2 was isolated from the rhizosphere soil of mangroves located within the Sanya River Mangrove Nature Reserve, Hainan Province, China.

The enrichment and isolation of AHL-degrading bacteria were performed following the procedures described in references (Vinoj et al., 2014). The AHL-degrading capability of the isolates was preliminarily screened using the cross-streak method with *Agrobacterium tumefaciens* KYC55 as the biosensor strain (Rosier and Bais, 2022). KYC55 carries a traI::lacZ transcriptional fusion and produces a blue pigment (indicating β-galactosidase activity) in response to AHLs. Putative QQ bacteria were streaked perpendicularly across the center of an LB agar plate, while the KYC55 biosensor was streaked horizontally across the plate, crossing the putative isolate. After incubation at 28 °C for 48 h, isolates that caused a visible reduction or absence of blue pigmentation in the biosensor strain were identified as potential AHL-degraders.

To quantitatively assess the QQ activity, the isolates were cultured in LB broth supplemented with synthetic C6-HSL (5 μM). After incubation, the supernatants were collected, filtered (0.22 μm pore size), and mixed with the biosensor strain. The residual AHL concentration was determined by measuring the optical density of the biosensor’s pigment production at 600 nm using a microplate reader (Tang et al., 2020).

Genomic DNA of the candidate QQ bacterium (designated as strain T2) was extracted using a standard SDS-based method. The 16S rRNA gene was amplified via polymerase chain reaction (PCR) using the universal primers 27F (5′-AGAGTTTGATCCTGGCTCAG-3′) and 1492R (5′-GGTTACCTTGTTACGACTT-3′). The PCR product was purified, cloned into the pMD™19-T vector (TaKaRa, Japan), and sequenced. The obtained full-length 16S rRNA sequence was compared with reference sequences in the GenBank database via BLAST algorithm, and phylogenetic trees were constructed using the neighbor-joining method in MEGA 7.0 software to accurately confirm the species-level taxonomic identity of strain T2 (Guo et al., 2018).

To chemically verify the degradation of AHLs, *B. licheniformis* T2 was cultured in LB medium containing 50 μM of specific AHLs (e.g., C6-HSL, C8-HSL, or 3-OH-C12-HSL). Culture supernatants were collected at different time intervals, mixed with an equal volume of acidified ethyl acetate, and vigorously vortexed. The organic phase was evaporated to dryness under a stream of nitrogen gas and reconstituted in 1 mL of methanol.

Only residual AHLs were quantified using a liquid chromatography-tandem mass spectrometry (LC-MS/MS) system equipped with an electrospray ionization (ESI) source operating in positive ion mode (Birmes et al., 2019). The degradation rate was calculated by comparing the peak areas of AHLs in the experimental group with those in the sterile control group.

### Screening and identification of CRAB clinical isolates with 3-OH-C12-HSL secretion

A total of ten non-duplicate CRAB isolates were selected from the de-identified, archived bacterial collection of the Department of Microbiology Laboratory at the First Affiliated Hospital of Hainan Medical University. These isolates were originally cultured from routine clinical specimens (including sputum, bronchoalveolar lavage fluid, and blood) collected retrospectively from January 2020 to December 2023, covering intensive care units (ICUs), neurosurgery, and respiratory medicine departments. The CRAB isolate exhibiting the highest 3-OH-C12-HSL production was selected for all subsequent mechanistic assays. As this study utilized only de-identified, previously collected isolates and did not involve prospective patient recruitment or interventions, the requirement for informed consent was waived by the Institutional Review Board. The study protocol was fully reviewed and approved by the Ethics Committee of Hainan Medical University (No. HYLL-2023-033). All experiments were conducted in accordance with the relevant guidelines and regulations.

Initial identification was performed using the VITEK^®^ 2 Compact system (bioMérieux, France). The minimum inhibitory concentration (MIC) was interpreted according to the latest Clinical and Laboratory Standards Institute (CLSI) guidelines. Species confirmation was performed via PCR amplification of the *blaOXA-51* gene, an intrinsic marker for *A. baumannii*. Screening for the abaI/abaR quorum-sensing locus was conducted as previously described (Tang et al., 2020). Isolates were defined as CRAB if they exhibited resistance to imipenem and meropenem. The antibiotic susceptibility profile of CRAB was determined using the broth microdilution method according to the Clinical and Laboratory Standards Institute (CLSI) guidelines. The MIC was defined as the lowest concentration of antibiotic that inhibited visible bacterial growth after 24 h of incubation at 37 °C. Antibiotics tested included cefepime, ceftazidime, meropenem, imipenem, levofloxacin, piperacillin/tazobactam, cefoperazone/sulbactam, tigecycline, and colistin.

This selection ensured the inclusion of CRAB strains possessing a functionally active quorum-sensing system. The primary autoinducer of CRAB, 3-OH-C12-HSL, was detected using the biosensor strain *Agrobacterium tumefaciens* KYC55. This reporter carries a traI-lacZ fusion, enabling specific detection of long-chain AHLs via β-galactosidase activity. Test isolates were cultured in LB broth at 37 °C for 18 h. Cell-free supernatants were obtained by centrifugation (12,000 × *g*, 10 min) and filtration (0.22 μm). A. tumefaciensKYC55 was cultured overnight at 28 °C in LB supplemented with 100 μg/mL tetracycline and 100 μg/mL spectinomycin. The assay was conducted in 96-well plates containing 50 μL of filtered supernatant, 50 μL of standardized KYC55 suspension (OD_600_ = 0.3), and 100 μL of LB medium with 40 μg/mL X-gal. Plates were incubated at 28 °C for 6–8 h. The reaction was terminated by adding 50 μL of 1 M Na_2_CO_3_. Absorbance was measured at 620 nm using a microplate reader. To confirm the specificity for 3-OH-C12-HSL, competitive inhibition assays were performed using synthetic 3-OH-C12-HSL (Sigma-Aldrich) as a reference standard. Isolates inducing a significant chromogenic response (>2-fold increase over background) were classified as 3-OH-C12-HSL producers.

### Genomic identification and validation of the ytnP gene

Genomic DNA (gDNA) was extracted from *Bacillus licheniformis* T-2 using the DNeasy Blood & Tissue Kit (Qiagen, Hilden, Germany) following the manufacturer’s protocol. The presence of *ytnP* was confirmed by PCR using specific primers: *ytnP*-F1 (5′-GCTGCTTGATTGCGAACTG-3′) and *ytnP*-R1 (5′-CGCTTGTTCGATTTCTCCG-3′). The amplicon was Sanger-sequenced (Macrogen Inc., Seoul, South Korea) and aligned to the assembled genome to resolve potential discrepancies. Sequence alignment of the amplified *ytnP* amplicon revealed a conserved HXHXDH catalytic motif characteristic of metallo-lactonases. Other putative hydrolase homologs retrieved from public databases lack complete catalytic domains and are predicted to exert negligible AHL-degrading activity (Xu et al., 2024).

### Phylogenetic analysis and protein structure prediction

Genomic sequences of YtnP homologs were retrieved from the NCBI database. Multiple sequence alignments were performed using ClustalW. A circular phylogenetic tree was constructed using the neighbor-joining method with 1,000 bootstrap replicates in MEGA 11.0 software. Nodes with bootstrap support values ≥0.55 were annotated with red dots proportional to their statistical confidence. The tertiary structure of YtnP was predicted via homology modeling using SWISS-MODEL, with the lactonase structure (PDB ID: 3EHB) as the template. The secondary structure was predicted using PSIPRED and JPred (Peng et al., 2021). Visualization of protein structures was performed using PyMOL. Sequence alignment additionally reveals unique variations in YtnP’s substrate pocket that favor binding to CRAB’s key signal 3-OH-C12-HSL.

### Molecular identification, expression and purification of recombinant YtnP

The presence of the *ytnP* gene in *Bacillus licheniformis* T-2 was confirmed by PCR using specific primers *ytnP*-F1 (5′-GCTGCTTGATTGCGAACTG-3′) and *ytnP*-R1 (5′-CGCTTGTTCGATTTCTCCG-3′), which were designed based on the conserved regions of the *ytnP* locus (GenBank: NZ_CP140161.1). The amplicon was verified by 1.0% agarose gel electrophoresis. Subsequently, the *ytnP* gene was PCR-amplified and cloned into the pCold™II vector via *Kpn* I/*Hind* III digestion, followed by transformation into *E. coli* BL21(DE3). Cultures were induced with 0.2 mM IPTG at 15 °C –16 °C for 24 h when the OD_600_ reached 0.4–0.6. Cells were harvested and lysed by sonication on ice. Recombinant YtnP was purified from the soluble supernatant by Ni^2+^-NTA affinity chromatography under native conditions using a linear imidazole gradient (20–500 mM) in 20 mM PBS (pH 7.0, 500 mM NaCl). The eluted protein was dialyzed against PBS, quantified by Bradford assay, and purity was verified by SDS-PAGE.

### Characterization of enzymatic activity and stability: effects of pH and temperature

The catalytic optimum of YtnP was assessed at 25 °C across a pH gradient of 6.0–8.0 using 25 μM 3-OH-C12-HSL as the substrate, buffered with 100 mM citrate-phosphate. To determine the optimal temperature, YtnP activity was measured at temperatures ranging from 0 °C to 100 °C. Thermal stability was assessed by pre-incubating the enzyme at different temperatures for 30 min, followed by residual activity measurement under standard conditions. For pH optima, the enzyme activity was tested in buffers with pH values ranging from 3.0 to 9.0 (citrate-phosphate buffer for pH 3.0–6.0, phosphate buffer for pH 6.0–8.0, and Tris-HCl buffer for pH 8.0–9.0). pH stability was evaluated by incubating YtnP in different buffers at 4 °C for 24 h, followed by activity measurement. One unit (U) of enzyme activity was defined as the amount of enzyme required to produce a detectable signal equivalent to the hydrolysis of 1 μmol of the substrate per minute.

### Biochemical characterization of YtnP

Thermostability was evaluated by pre-incubating the enzyme at 0 °C –100 °C for 30 min prior to activity assay. The optimal pH was determined across pH 3.0–9.0 using appropriate universal buffer systems (citrate-phosphate for pH 3.0–6.0, phosphate for 6.0–8.0, Tris-HCl for 8.0–9.0). Metal ion dependency was assessed by supplementing the reaction buffer with 1 mM of various divalent cations (Ni^2+^, Mn^2+^, Ca^2+^, Mg^2+^, Zn^2+^, Fe^2+^) or 5 mM EDTA. Residual substrate concentrations were quantified via high-performance liquid chromatography (HPLC).

### RNA extraction and quantitative real-time PCR (qRT-PCR)

Total RNA was extracted from CRAB cells treated with or without YtnP using TRIzol reagent. cDNA was synthesized using PrimeScript RT Master Mix. qRT-PCR was performed using SYBR Premix Ex Taq on a CFX96 Touch system. The relative expression levels of outer membrane protein genes (*ompA*, *csuA/B*) were normalized to the housekeeping gene *gyrB* and calculated using the 2^–ΔΔ*Ct*^ method.

### Biofilm disruption assays and microscopy

Carbapenem-resistant *Acinetobacter baumannii* biofilms were established in 12-well plates for 24 h. Mature biofilms were treated with purified YtnP. Biofilm viability was assessed using a Live/Dead BacLight kit (SYTO 9/propidium iodide) and visualized via confocal laser scanning microscopy (CLSM). Quantitative structural parameters, including biomass (μm^3^/μm^2^) and average/maximum thickness (μm), were calculated using COMSTAT software (MATLAB-based). Thresholding was performed automatically via the COMSTAT GUI to distinguish live (green) and dead (red) biomass fractions.

### Evaluation of bactericidal activity against planktonic cells

To determine whether YtnP exerts direct bactericidal effects, planktonic growth assays were performed. Overnight cultures of CRAB were diluted 1:100 into fresh LB broth containing either purified YtnP (final concentration: 50 μg/mL, corresponding to 2.5 U/mL) or an equivalent volume of PBS (vehicle control). Cultures were incubated at 37 °C with shaking, and optical density (OD_600_) was measured hourly for 12 h using a microplate reader. Additionally, aliquots were collected at 0, 6, and 12 h for colony-forming unit (CFU) enumeration. Samples were serially diluted in PBS and plated onto LB agar plates in triplicate. Plates were incubated at 37 °C for 18–24 h, and viable counts were calculated. No significant differences in OD_600_ growth curves or CFU viability were observed between YtnP-treated and control groups, demonstrating that YtnP exhibits no direct bactericidal or growth-inhibitory effect on planktonic CRAB at the working concentration used for anti-virulence and antibiofilm assays.

### *In vivo* virulence assessment using the *Galleria mellonella* model

Preparation of heat-inactivated YtnP control. To exclude the possibility that the observed protective effects were attributable to nonspecific protein binding or impurities rather than enzymatic activity, a heat-inactivated YtnP control was prepared. Purified recombinant YtnP (50 μg/mL) was incubated at 95 °C for 15 min to fully abolish lactonase activity. The complete loss of enzymatic activity was confirmed by the failure to degrade 3-OH-C12-HSL under standard assay conditions prior to the inoculation experiments.

*In vivo* virulence assessment using the *Galleria mellonella* model. The protective efficacy of YtnP against CRAB virulence was evaluated using a *Galleria mellonella* larval model (Ménard et al., 2021). Stationary-phase supernatants (18 h, 37 °C) from a carbapenem-resistant clinical isolate (MIC > 16 mg/L) were clarified via centrifugation (8,000 × *g*, 15 min) followed by 0.22-μm filtration to ensure the complete removal of viable bacteria. The filtered supernatants were divided into three aliquots: (i) untreated control; (ii) supplemented with active YtnP (final concentration: 50 μg/mL, corresponding to 2.5 U/mL); and (iii) supplemented with heat-inactivated YtnP (final concentration: 50 μg/mL), total protein load and buffer composition were matched to the active-YtnP group. All aliquots were incubated at 37 °C for 1 h to allow hydrolysis of 3-OH-C12-HSL. Groups (*n* = 10 larvae per group) were established: (1) CRAB + YtnP, (2) CRAB + inactivated YtnP, (3) CRAB (vehicle control), and (4) Negative control (sterile 0.9% NaCl). Final-instar larvae (Vanderhorst Wholesale, Inc.), weighing 300 ± 50 mg, were surface-sterilized with 70% ethanol. Subsequently, 2 μL of the prepared supernatants was injected into the hemocoel via the last left proleg using a 10-μL Hamilton syringe. Following injection, larvae were incubated in sterile Petri dishes in the dark at 25 °C without feeding.

### Statistical analysis

All experiments were performed in triplicate. Data are presented as means ± standard deviation (SD). Statistical significance was determined using Student’s *t*-test for pairwise comparisons or one-way ANOVA followed by Tukey’s *post hoc* test for multiple comparisons. Larval survival was monitored at 2-h intervals for 36 h. Death was defined as the absence of movement upon tactile stimulation combined with visible cuticular melanization. Survival curves were generated using the Kaplan–Meier method, and differences between groups were compared using the log-rank (Mantel–Cox) test. A *P*-value < 0.05 was considered statistically significant.

## Results

### Phylogenetic analysis and protein structure prediction of YtnP homologs

A circular phylogenetic tree was constructed to elucidate the evolutionary relationships among YtnP homologs from diverse bacterial species (Figure 1A). The tree topology revealed distinct clades corresponding to different taxonomic groups. Nodes with bootstrap support values ≥0.55 are indicated by red dots, where dot size correlates positively with bootstrap scores. Notably, *Bacillus licheniformis* T-2 (highlighted in magenta) occupied a unique branch and clustered closely with other *Bacillus* spp. (e.g., *Bacillus altitudinis*NC3, *Bacillus atrophaeus*UCMB5137), while remaining phylogenetically distant from non-*Bacillus* taxa (e.g., *Pseudonobacillus rhizosphaerae*, *Lysinibacillus fusiformis*, *Arthrobacter* sp.). Furthermore, several YtnP variants (e.g., AiiA, MomL, AhiS, AiiT, AiiB, AhD) from taxonomically diverse bacteria (e.g., *Bacillus thuringiensis*, Bacteroidetes bacterium, *Mammaliicoccus sciuri*) formed separate lineages, suggesting potential functional or evolutionary divergence. Bootstrap values ranged from 0.55 to 1.00, confirming the robustness of the observed branching patterns. Key nodes, such as the clade containing *Bacillus* YtnPs, exhibited strong statistical support (bootstrap ≥ 0.89).

**FIGURE 1:**
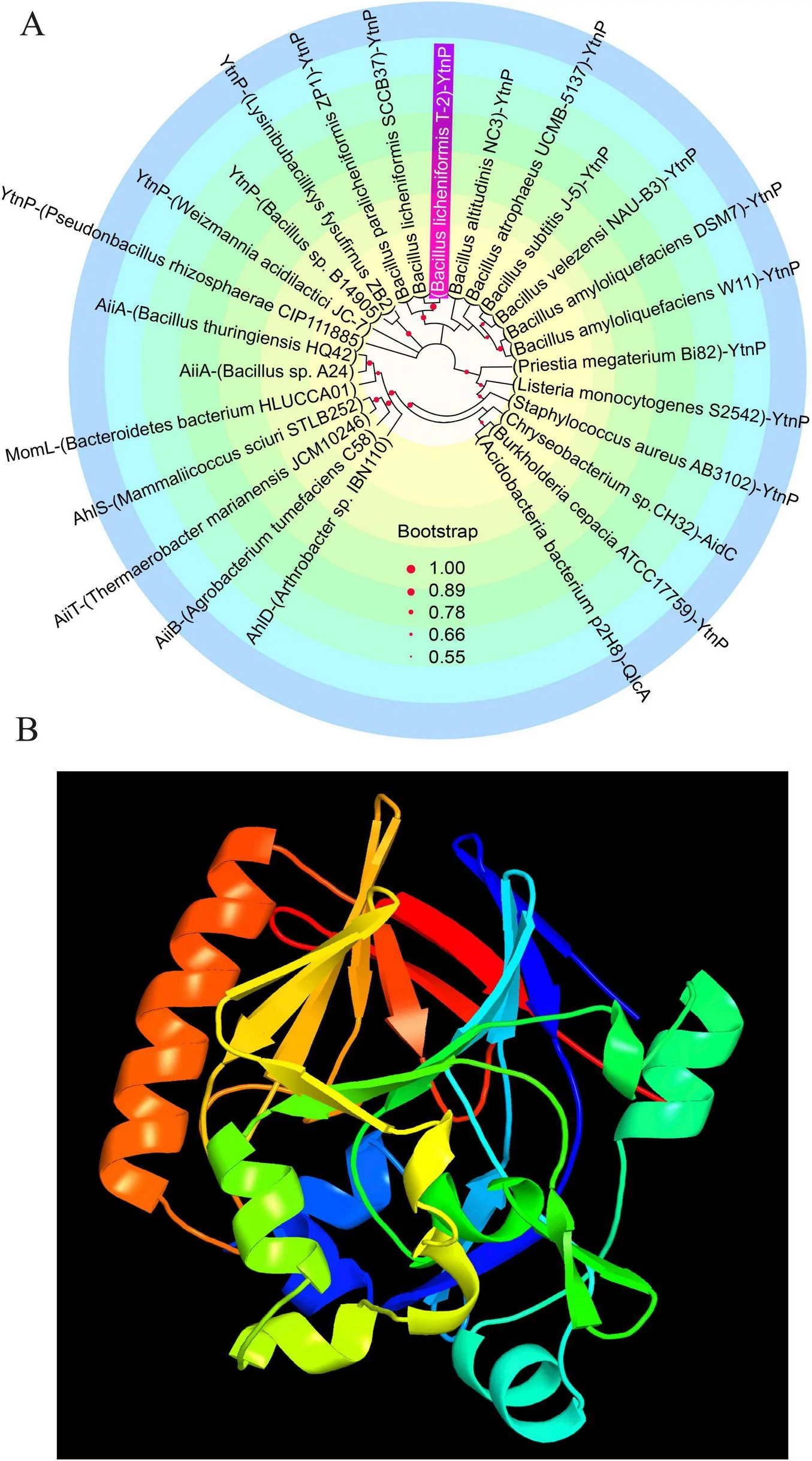
Phylogenetic analysis and protein structure modeling of YtnP. **(A)** Phylogenetic tree of YtnP homologs from diverse bacterial species, constructed via the neighbor-joining method (MEGA 11.0). Bootstrap values (≥50%) are indicated at nodes. The YtnP sequence from this study is highlighted in pink. **(B)** Three-dimensional structural model of YtnP protein.

The tertiary structure of YtnP was predicted using SWISS-Model with the lactonase structure (PDB ID: 3EHB) as a template, exhibiting 49.5% sequence identity (confidence > 95%). The ribbon diagram revealed a compact globular fold characteristic of *B. licheniformis* T-2 YtnP (Figure 1B). Secondary structure analysis showed a balanced distribution of α-helices (orange, yellow) and β-sheets (red, cyan, blue), interconnected by loop regions (green). Notably, the protein surface is predominantly composed of hydrophilic α-helices, encapsulating a core rich in β-sheets. This architectural arrangement suggests the presence of distinct functional domains, potentially harboring catalytic or substrate-binding sites. This model provides a robust structural framework for elucidating the enzymatic mechanism and protein-protein interactions of YtnP homologs.

### Molecular identification and biochemical characterization of YtnP

The presence of the *ytnP* gene in *Bacillus licheniformis* T-2 was confirmed by PCR using specific primers (*ytnP*-F1/R1) and genomic DNA as the template. Agarose gel electrophoresis revealed a single, distinct amplicon of approximately 950 bp (Lane 4, Figure 2A), which corresponds to the expected size of the full-length *ytnP* gene. No amplification products were detected in the negative controls (Lanes 1–3), which included reactions lacking template DNA or primers, confirming the specificity of the assay.

**FIGURE 2:**
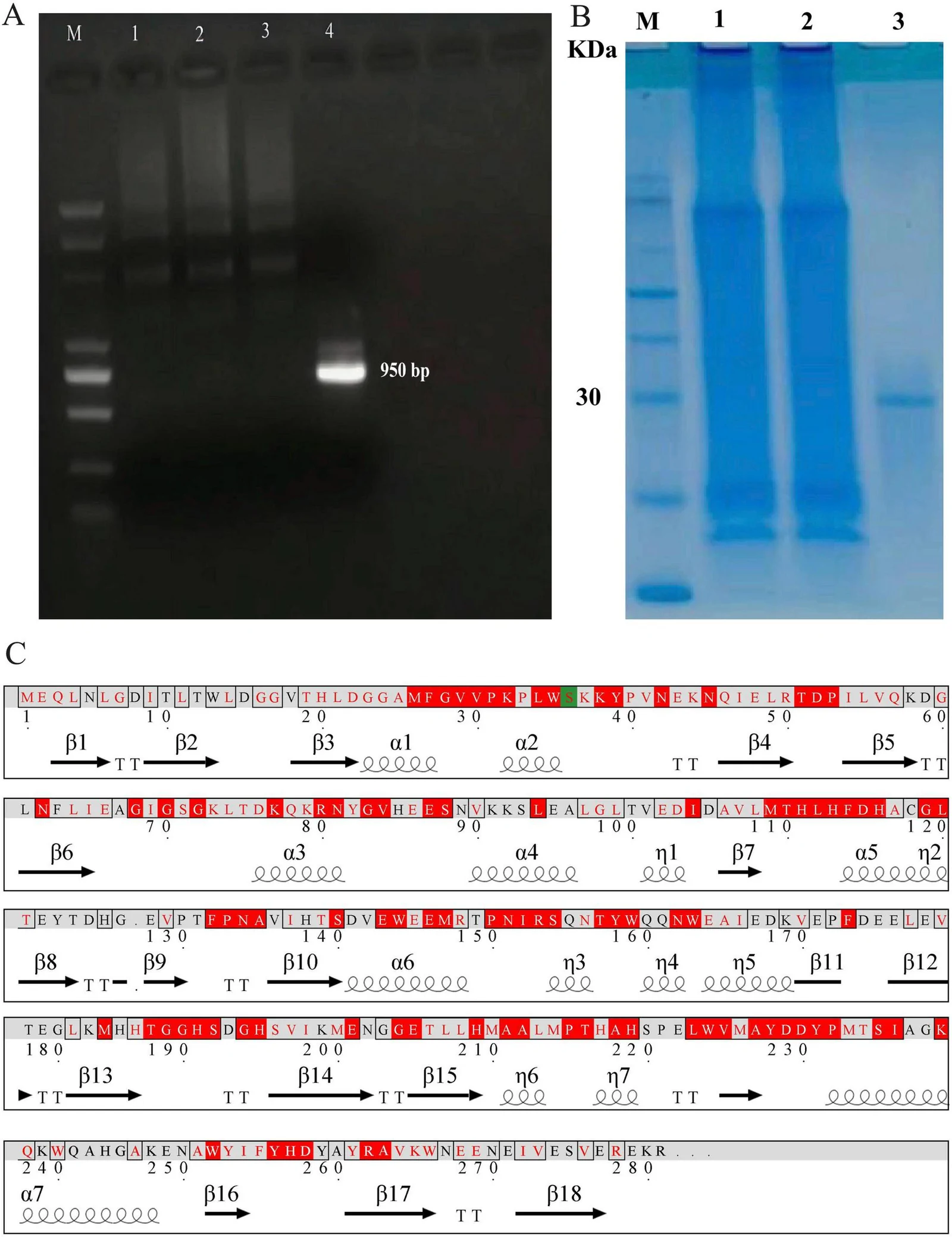
Gene cloning and sequence analysis of YtnP. **(A)** Agarose gel electrophoresis of the full-length *ytnP* gene PCR product. Using genomic DNA from *Bacillus licheniformis* T-2 as the template and specific primers *ytnP*-F1/ytnP-R1 for amplification, a 950 bp fragment corresponding to the full-length *ytnP* gene was obtained (Lane 4). Lane 1: Negative control with ddH2O replacing template DNA; Lane 2: Negative control without template DNA; Lane 3: Blank control without primers; M: DNA molecular weight marker. **(B)** SDS-PAGE analysis of YtnP enzyme from *Bacillus licheniformis* T-2. M: Protein marker; Lane 1: Cytoplasmic fraction of cells harboring the pCold™II plasmid; Lane 2: Cytoplasmic fraction of cells harboring the pCold-II-*ytnP* plasmid; Lane 3: Purified YtnP protein, with an approximate molecular weight of 30 kDa. **(C)** Amino acid sequence alignment of YtnP. The *ytnP* gene encodes a total of 280 amino acids.

The expression and purification of the recombinant YtnP protein were evaluated by SDS-PAGE (Figure 2B). Total cell lysates from *Bacillus licheniformis* T-2 harboring the empty pCold™II vector exhibited multiple protein bands (Lane 1), whereas cells transformed with the pCold-II-ytnP construct displayed an additional multiple protein bands (Lane 2). Following affinity chromatography, a single dominant band corresponding to the predicted molecular weight of YtnP (30 kDa) was observed (Lane 3), indicating successful overexpression and high-purity isolation.

The secondary structure of YtnP was predicted using ab initioalgorithms (PSIPRED and JPred) to delineate α-helices, β-sheets, and random coils. As illustrated in Figure 2C, the 280-amino-acid sequence of YtnP is organized into seven α-helices (α1–α7), eighteen β-strands (β1–β18), and seven η-regions (η1–η7, representing random coils). The protein exhibits a balanced α/β architecture, with the N-terminal region primarily composed of α1/α2 and β1–β5, and the C-terminal region characterized by α6/α7 and β13–β18. The terminal ends (residues 1–10 and 270–280) are predominantly unstructured. Notably, specific residues highlighted are predicted to be involved in functional motifs, potentially corresponding to catalytic or ligand-binding sites. Multiple amino acid sequence alignment of YtnP in *Bacillus licheniformis* T-2 revealed the presence of a zinc-binding conserved domain HXHXDH. Therefore, it was speculated that the YtnP in *Bacillus licheniformis* T-2 composed of 280 amino acid residues belonging to the same category as metallo-β-lactamase superfamily.

### Biochemical characterization of YtnP: thermostability and pH-dependent catalytic profiles

To characterize the thermal properties of YtnP, its activity and stability were evaluated across a temperature range of 0–100 °C using 3-OH-C12-HSL as substrate (Figure 3A). The activity of YtnP (green line) increased gradually with temperature, reaching a peak at 45 °C, before declining sharply at temperatures >45 °C. In contrast, the stability of YtnP (red line) remained relatively stable up to ∼40 °C but decreased progressively at higher temperatures (≥45 °C). Notably, the temperature optimum for activity (37 ∼ 45 °C) coincided with a critical threshold for stability, suggesting a trade-off between catalytic efficiency and thermostability in this range.

**FIGURE 3:**
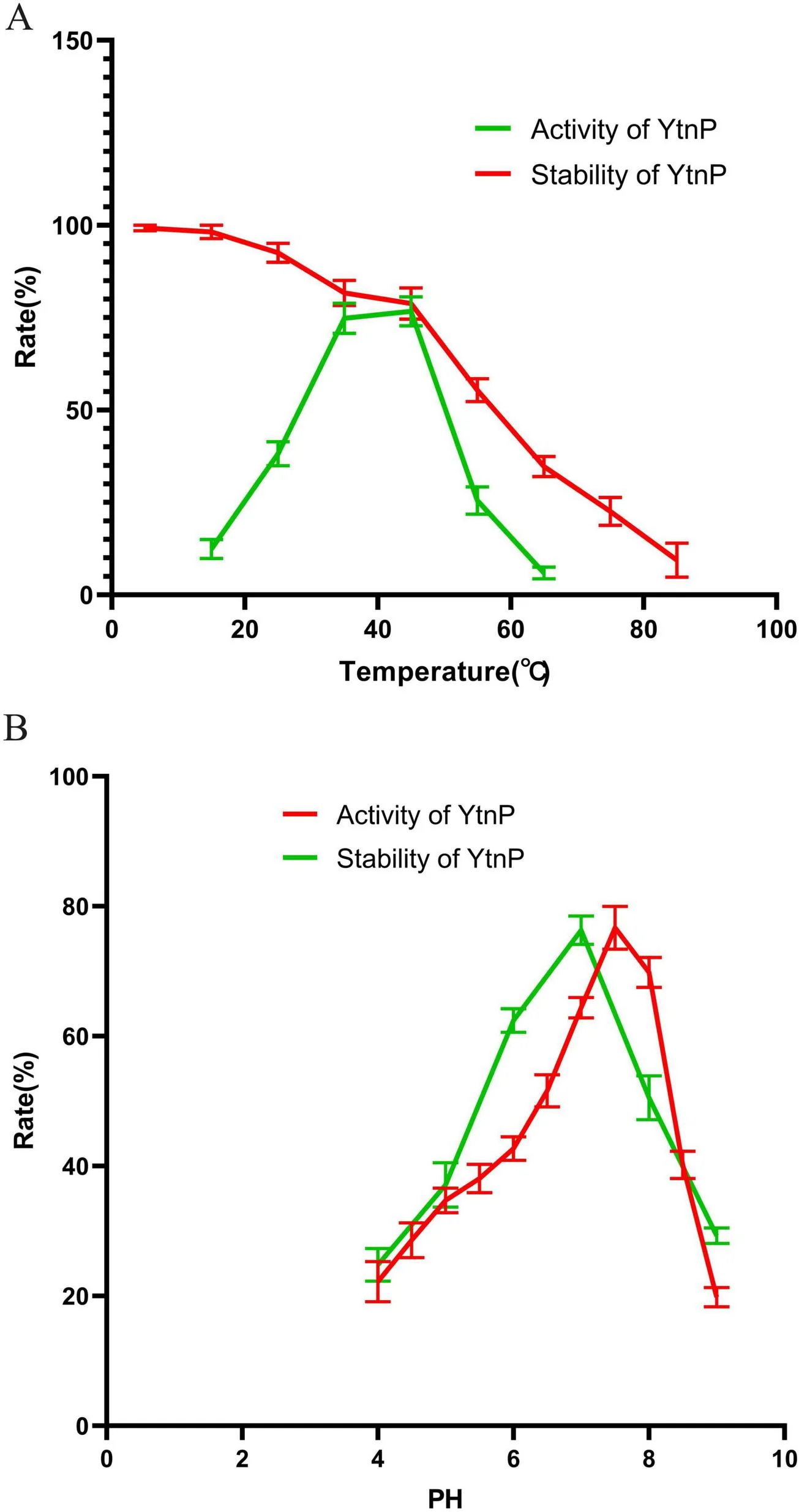
Effects of temperature and pH on YtnP activity and stability. **(A)** Effect of temperature on the activity and stability of YtnP using 3-OH-C12-HSL as substrate. **(B)** Effect of pH on the activity and stability of YtnP using 3-OH-C12-HSL as substrate.

The effect of pH on YtnP activity and stability was investigated over the range of pH 3.0–9.0 using 3-OH-C12-HSL as substrate (Figure 3B). Both activity (red line) and stability (green line) exhibited a bell-shaped profile, with optimal performance at pH ∼7.0–7.5. At pH < 5.0 or >8.0, both activity and stability declined significantly, indicating a narrow pH tolerance window. Specifically, YtnP retained >60% of its maximal activity and stability at pH 6.0–8.0, whereas at pH 4.0 or 9.0, these values dropped below 40%. This pH-dependent behavior highlights the enzyme’s preference for neutral-to-slightly-alkaline environments.

### Metal ion dependency and structural determinants of YtnP activity

The effects of divalent metal ions and the chelating agent EDTA on YtnP lactonase activity were investigated using 3-OH-C12-HSL as the substrate (Figure 4A). Ni^2+^, Mn^2+^, Ca^2+^, or Mg^2+^ did not significantly alter its activity. In contrast, Zn^2+^, Fe^2+^, and EDTA exerted substantial inhibitory effects, reducing the relative activity by 42.2%, 37.7%, and 69.3%, respectively. Unlike most canonical AHL lactonases that require exogenous Zn^2+^ for catalytic activation, YtnP contains a conserved HXHXDH metalloenzyme motif but is suppressed by excess external Zn^2+^. This implies that the pre-assembled catalytic metal center within the rigid active pocket is structurally saturated, and additional Zn^2+^ disrupts substrate binding and catalytic geometry.

**FIGURE 4:**
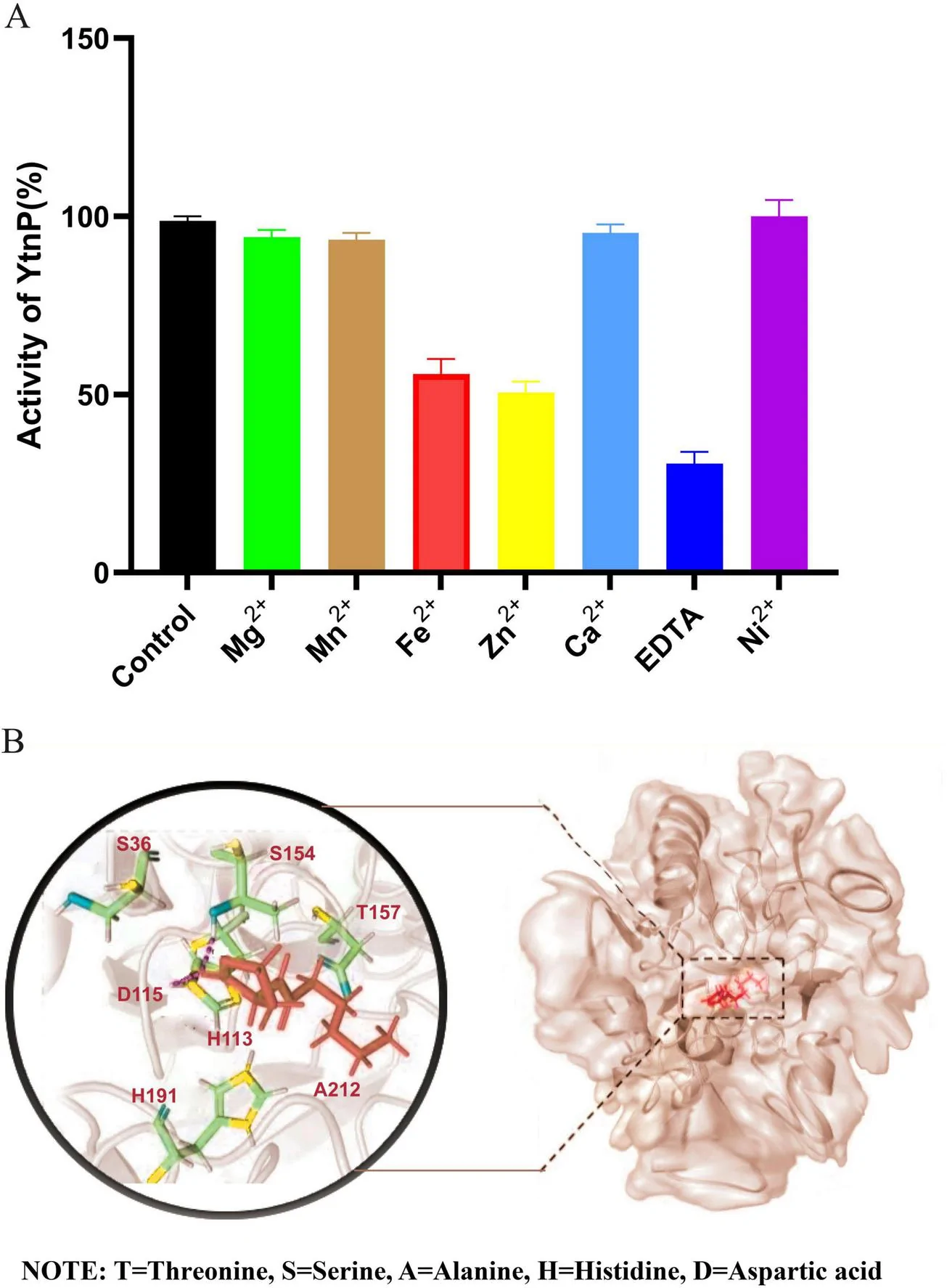
Effects of metal ions and molecular docking of YtnP with AHL. **(A)** Relative activity of YtnP after pre-incubation with 5 mM metal ions (Zn^2+^, Mn^2+^, Ni^2+^, Ca^2+^, Mg^2+^, Fe^2+^) or EDTA in 50 mM Tris-HCl (pH 7.5, 25 °C, 5 min). Activity was measured with 25 μM 3-OH-C12-HSL at 45 °C (pH 7.5). Control: no metal ions/EDTA. **(B)** Molecular docking model of YtnP with 3-OH-C12-HSL. Key catalytic residues (S36, H113, H191, D115, S154, T157, A212) at the active site are shown.

To elucidate the molecular basis of metal-dependent inhibition, the 3D structural model of YtnP was analyzed (Figure 4B). A magnified view of the active site revealed a cluster of evolutionarily conserved residues–S36, H113, D115, S154, T157, H191, and A212–strategically positioned to form a putative catalytic pocket. The pocket is predominantly lined with polar residues, suggesting that metal coordination occurs via the hydroxyl (-OH) groups of Ser/Thr or the carboxyl (-COOH) groups of Asp. Molecular docking simulations with 3-OH-C12-HSL confirmed that these residues are spatially poised to interact with the lactone ring. Notably, the presence of two histidines (H113, H191) flanking an aspartic acid (D115) mirrors the canonical metal-binding motifs found in metallo-hydrolases, implying that these residues may facilitate catalysis through proton transfer or electrostatic stabilization of the transition state.

### Phenotypic analysis of CRAB drug resistance and regulation of key outer membrane proteins by YtnP

The antimicrobial susceptibility profile of the clinical CRAB isolate was determined using the broth microdilution method (Figure 5A). The strain exhibited extensive drug resistance, with MICs exceeding the clinical breakpoints for all tested carbapenems (meropenem > 16 mg/L; imipenem > 16 mg/L), cephalosporins (cefepime > 32 mg/L; ceftazidime > 64 mg/L), and β-lactam/β-lactamase inhibitor combinations (piperacillin/tazobactam > 128 mg/L; cefoperazone/sulbactam > 64 mg/L). Resistance was also observed for levofloxacin (MIC > 8 mg/L). In contrast, the isolate remained susceptible to the last-resort antibiotics tigecycline (MIC = 2 mg/L) and colistin (MIC = 0.5 mg/L), indicating that these agents retain potent activity against this CRAB strain.

**FIGURE 5:**
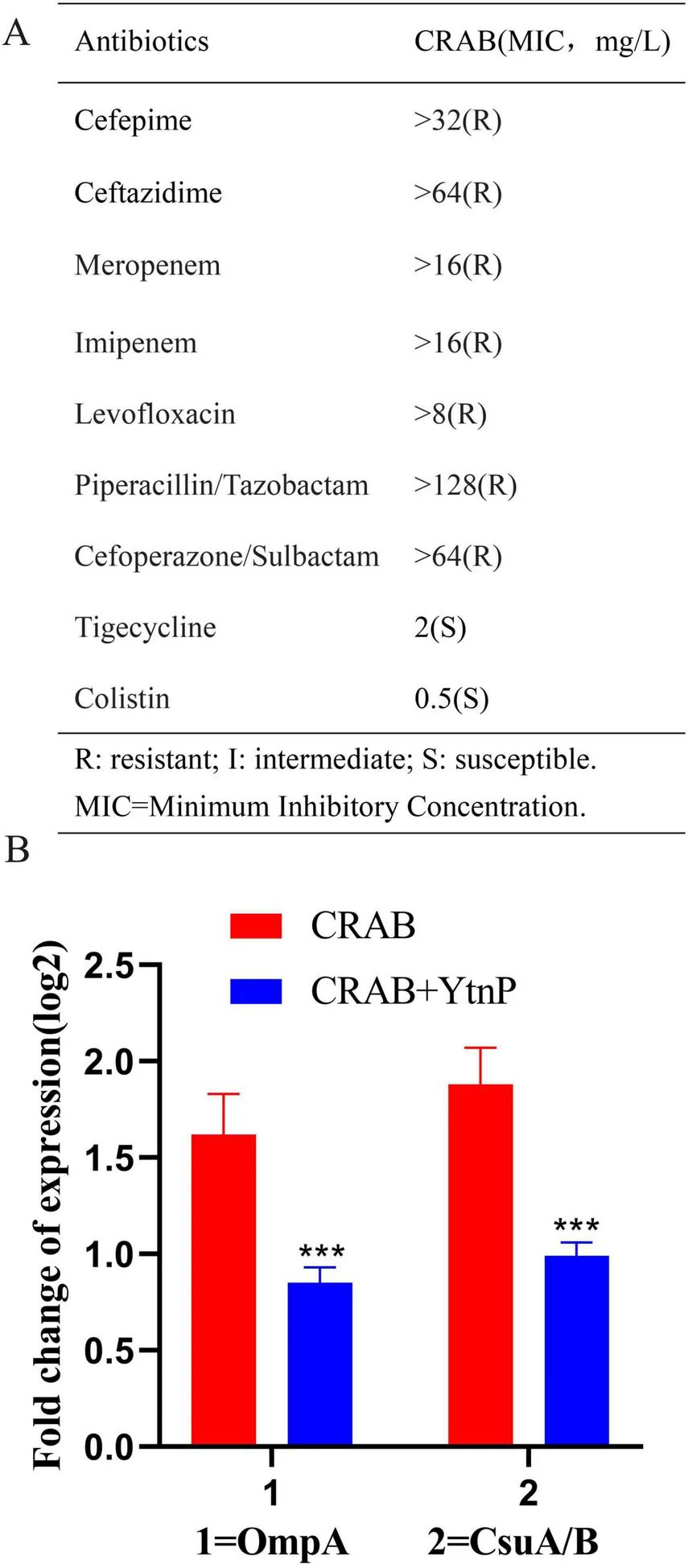
Antibiotic susceptibility of CRAB and YtnP-mediated regulation of biofilm/virulence genes. **(A)** The MICs of antibiotics against clinical CRAB isolates. **(B)** Quantitative real-time PCR analysis of *ompA* and *csuA/B* expression in CRAB treated with 50 μg/mL YtnP for 6 h. Data were analyzed using unpaired Student’s *t*-test for two-group comparison. ***p < 0.001 vs. untreated control group.

To elucidate the impact of YtnP on the outer membrane integrity of CRAB, the transcriptional levels of two key outer membrane proteins, OmpA and CsuA/B, were quantified via qRT-PCR (Figure 5B). Compared to the untreated control, treatment with YtnP resulted in a significant downregulation of both genes. Specifically, *ompA* expression was attenuated by approximately 50.0% (log2 fold change decreased from 1.6 ± 0.2 to 0.8 ± 0.1, *p* < 0.001), while *csuA/B* expression was reduced by 52.6% (log2 fold change decreased from 1.9 ± 0.3 to 0.9 ± 0.1, *p* < 0.001). These findings suggest that YtnP disrupts the transcriptional regulation of essential outer membrane proteins, which may compromise outer membrane stability, thereby enhancing antibiotic permeability or interfering with the assembly of virulence-associated structures.

### Attenuation of CRAB pathogenicity: YtnP induces biofilm eradication and interferes with QS signaling

The impact of YtnP on CRAB biofilm architecture was assessed using confocal laser scanning microscopy (CLSM) following live/dead staining (Figures 6A, B). Untreated control biofilms exhibited a dense, three-dimensional reticulated structure with robust green fluorescence (SYTO 9), indicating a mature biofilm populated by metabolically active cells. In stark contrast, YtnP treatment induced significant disruption of the extracellular matrix and a marked loss of structural integrity. This fluorescence shift reflected structural collapse of the biofilm extracellular matrix and microenvironmental stress-induced membrane damage in embedded cells, rather than direct bactericidal killing by YtnP, which is consistent with the unaffected viability of planktonic CRAB. These visual observations corroborate the quantitative reductions in total biomass and average thickness (Figures 6C, D). YtnP induced profound structural collapse of mature biofilms, characterized by marked loss of 3D architecture and a shift toward PI-dominant fluorescence. This shift is consistent with ECM disassembly and the loss of matrix-integrity-mediated cytoprotection, rather than direct bactericidal activity–a conclusion supported by the absence of CFU reduction in parallel planktonic assays.

**FIGURE 6:**
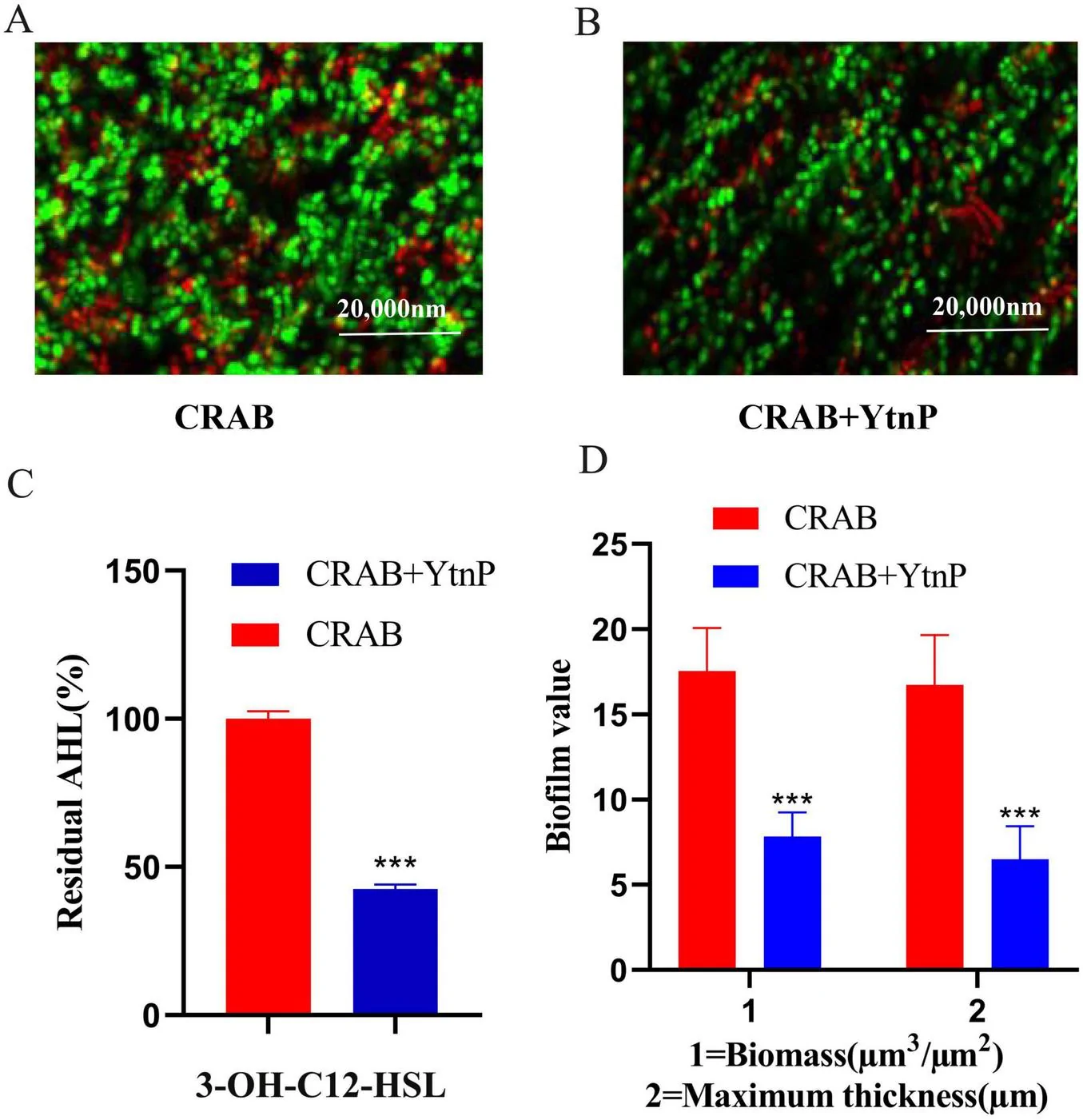
YtnP inhibits CRAB biofilm and quorum sensing signaling. **(A)** Confocal micrographs of mature CRAB biofilms (live/dead staining: green/red fluorescence). **(B)** Biofilms after YtnP treatment (50 μg/mL, 24 h). Reduced green fluorescence indicates fewer viable bacteria and thinner biofilm structure. **(C)** Hydrolysis efficiency of YtnP against CRAB-secreted 3-OH-C12-HSL (N-(3-hydroxy-dodecanoyl)-L-homoserine lactone). **(D)** Biofilm biomass (μm^3^/μm^2^) and maximum thickness (μm) quantified by COMSTAT. Quantitative data are shown as mean ± SD of independent biological replicates. Unpaired two-tailed Student’s *t*-test was used for comparisons between the CRAB group and CRAB + YtnP group. ***p < 0.001 vs. control group.

The quorum sensing-quenching activity of YtnP was evaluated by quantifying the degradation of the autoinducer 3-OH-C12-HSL via HPLC (Figure 6C). Compared to the untreated control, which maintained high baseline levels of the signaling molecule, treatment with YtnP resulted in a significant reduction of residual 3-OH-C12-HSL to approximately 40.5% of the initial concentration (corresponding to a 59.5% degradation efficiency, *p* < 0.001). These findings demonstrate that YtnP effectively hydrolyzes the key QS signal in CRAB, thereby disrupting bacterial communication and validating its anti-virulence potential.

The disruptive effect of YtnP on the biofilm ultrastructure was further validated by COMSTAT analysis, which provides quantitative metrics for biomass and thickness (Figure 6D). Compared to the untreated control, YtnP disrupted mature biofilms, reducing biomass by 55.56% (from 18.0 ± 2.9 to 8.0 ± 1.7 μm^3^/μm^2^; *p* < 0.001) and maximum thickness by 61.76% (from 17.0 ± 2.5 to 6.5 ± 1.8 μm; *p* < 0.001). These quantitative findings confirm that YtnP causes a severe reduction in the biofilm’s overall mass and structural density, which is consistent with the disruptive morphology observed via CLSM.

### Protective efficacy of YtnP against CRAB metabolite toxicity in a *G. mellonella* model

The *in vivo* detoxifying potential of YtnP was evaluated using a *Galleria mellonella* model (Figure 7). Larvae injected with sterile 0.9% NaCl maintained almost 100% survival, confirming the biosafety of the vehicle control. In contrast, inoculation with CRAB metabolites resulted in rapid lethality, with the survival rate plummeting to 16.67% within 12 h and 10.00% within 36 h. Similarly, the group treated with heat-inactivated YtnP also exhibited a sharp decline in survival, dropping to 16.67% at 12 h and 10.00% at 36 h. In contrast, supplementation with active YtnP markedly rescued larval survival, reaching 80.00% at 12 h and 63.33% at 36 h. This protection persisted throughout the assay; by 36 h, the survival rate in the YtnP-treated group (63.33%) was nearly sixfold higher than that of the untreated CRAB control (10.00%).

**FIGURE 7:**
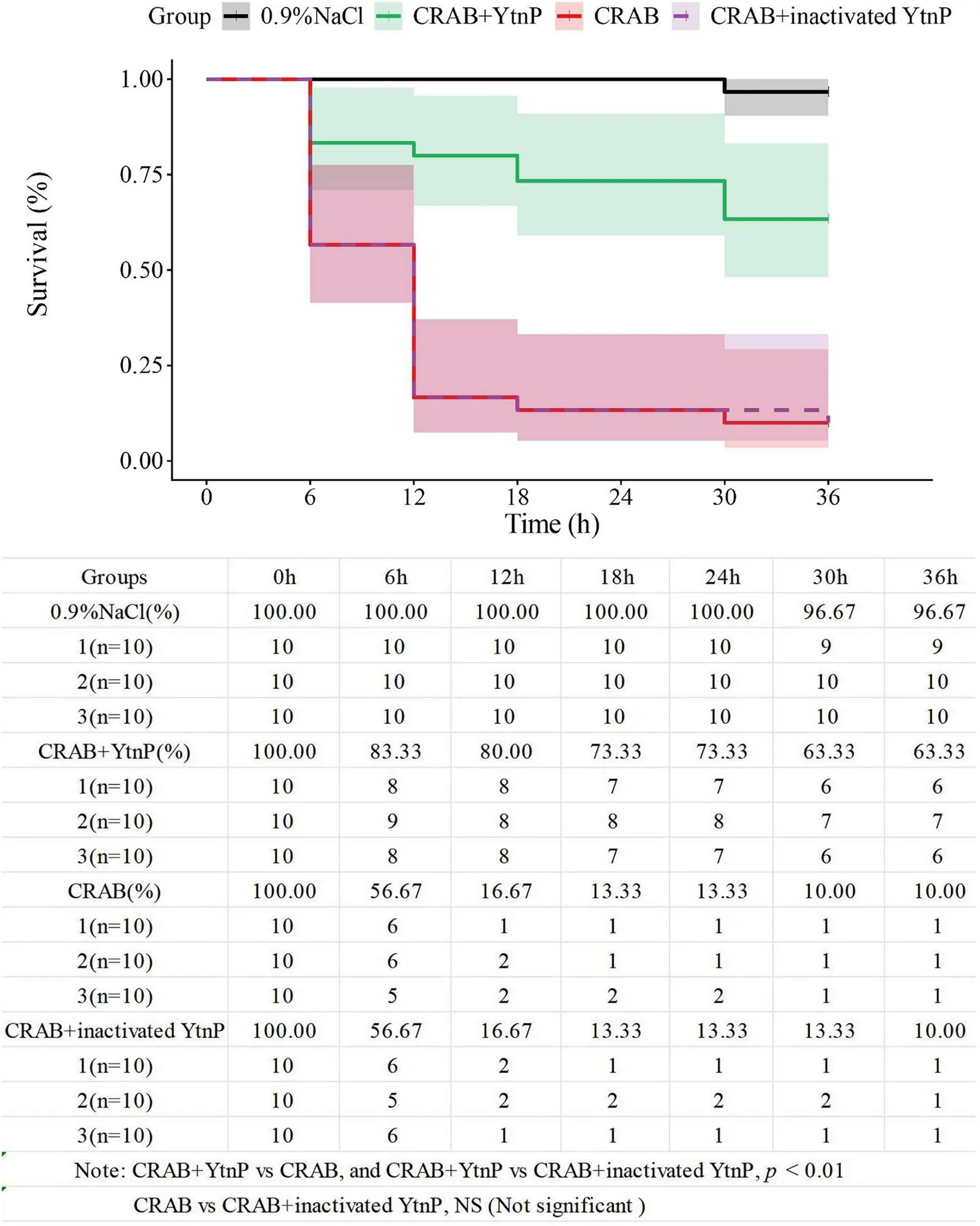
Kaplan–Meier survival curves of *Galleria mellonella* larvae challenged with cell-free CRAB culture supernatant, supernatant plus active YtnP, supernatant plus heat-inactivated YtnP, and sterile 0.9% NaCl as the negative control. Larval survival was recorded every 2 h for a total of 36 h.

Statistical analysis revealed that YtnP treatment significantly enhanced larval survival. The survival rates in the CRAB + YtnP group were significantly higher than those in both the CRAB-only group and the CRAB + inactivated YtnP group (both *p* < 0.01). No statistically significant difference was observed between the CRAB-only group and the CRAB + inactivated YtnP group (NS). These findings demonstrate that YtnP effectively mitigates the pathogenicity of CRAB metabolites, thereby rescuing host viability.

### YtnP exerts no direct bactericidal activity

To determine whether YtnP possesses direct bactericidal activity against CRAB, planktonic growth assays were performed (Figure 8). Overnight CRAB cultures were diluted 1:100 into fresh LB broth supplemented with either purified YtnP (final concentration: 50 μg/mL, corresponding to 2.5 U/mL) or an equal volume of PBS (vehicle control). Cultures were incubated at 37 °C with continuous shaking, and optical density (OD_600_) was measured hourly for 12 h using a microplate reader. Additionally, aliquots were collected at 0, 6, and 12 h for colony-forming unit (CFU) enumeration: samples were serially diluted in PBS, plated onto LB agar in triplicate, and incubated at 37 °C for 18–24 h prior to viable count calculation.

**FIGURE 8:**
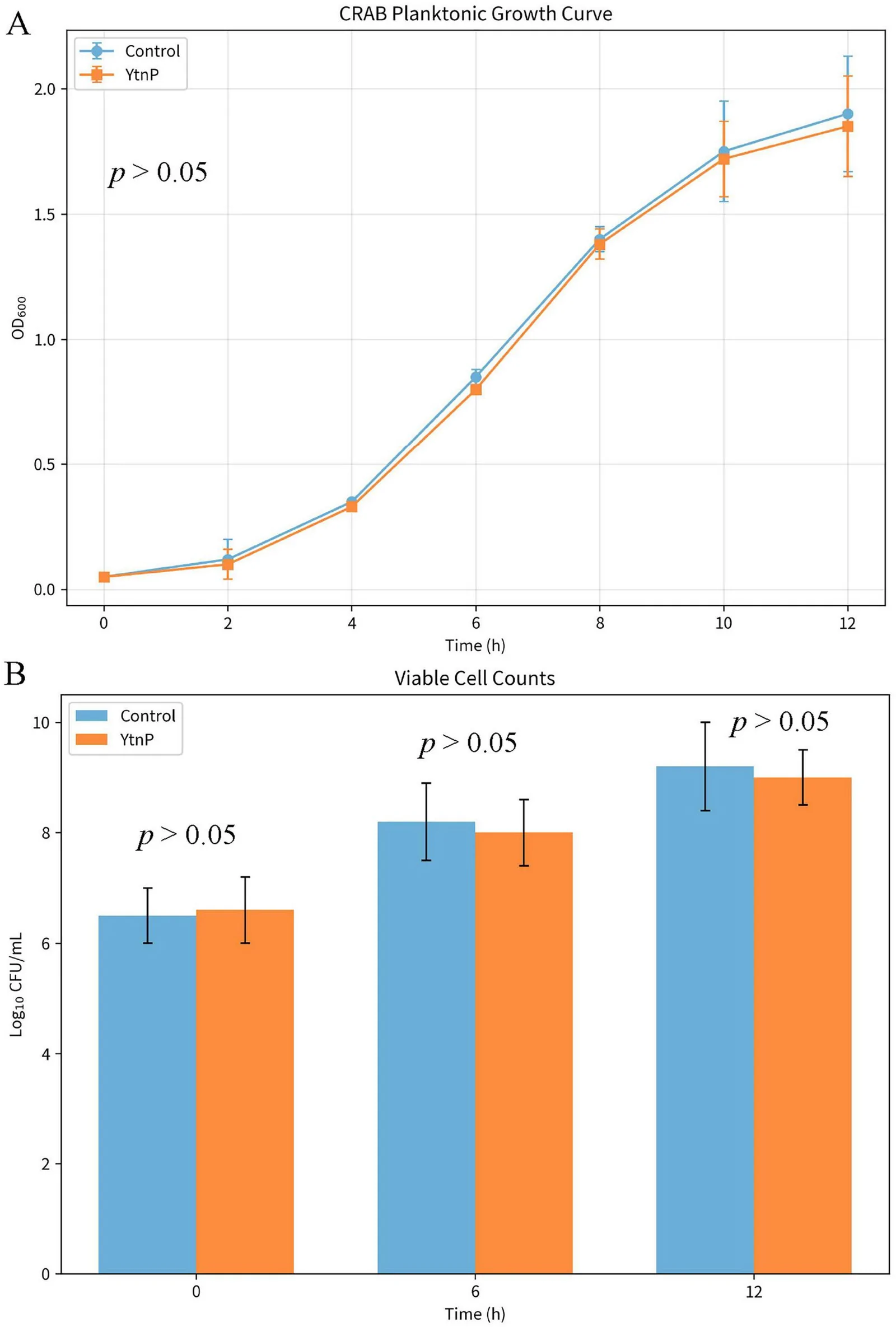
YtnP exerts no direct bactericidal effect on CRAB planktonic growth. CRAB was cultured in LB broth with or without 50 μg/mL YtnP. No significant differences in OD_600_ **(A)** or CFU **(B)** counts were observed between the treated and control groups over 12 h (*p* > 0.05), confirming YtnP does not inhibit planktonic viability.

No statistically significant differences were observed in OD_600_ values (Figure 8A) or CFU counts (Figure 8B) between the YtnP-treated group and the control group at any tested time point (*p* > 0.05). These results confirm that YtnP does not inhibit the growth or viability of CRAB planktonic cells at the concentrations applied in subsequent anti-virulence assays, indicating that its biofilm-disrupting and virulence-attenuating effects are independent of direct bactericidal activity.

The conclusion that YtnP lacks bactericidal or bacteriostatic activity against CRAB is supported by two independent quantitative readouts: hourly OD_600_ growth monitoring and triplicate CFU enumeration across multiple time points, alongside visual live/dead staining evidence of intact biofilm populations. No significant reduction in viable bacteria was detected in either planktonic cultures or mature biofilms following YtnP treatment.

## Discussion

Prior works have confirmed that YtnP-type lactonases are capable of degrading the key *A. baumannii* quorum-sensing signal 3-OH-C12-HSL. In this study, we identified a novel mangrove rhizosphere-derived YtnP from *Bacillus licheniformis* T2, further provided full biochemical characterization of its catalytic properties, and systematically verified its dual activities of disrupting mature CRAB biofilms and alleviating CRAB metabolite-induced host lethality in the *Galleria mellonella* model. These findings reinforce the feasibility of targeting bacterial communication rather than bacterial viability as a strategy to attenuate hyper-resistant pathogens with minimal selective pressure (Morris et al., 2019; Tay and Yew, 2013).

### YtnP targets a central QS node in CRAB pathogenesis

In CRAB, the AbaI/AbaR circuit represents the best-characterized LuxI/LuxR-type system, in which 3-OH-C12-HSL coordinates a regulon tied to surface colonization, motility, and biofilm maturation (Law and Tan, 2022; Vijayakumar and Thirunanasambandham, 2021). Key structural determinants of biofilm integrity–particularly the *csuA/BABCDE* pilus system and broadly important outer membrane factors such as OmpA–are downstream of, or modulated through, this QS landscape (Barati et al., 2024). Enzymatic degradation of 3-OH-C12-HSL by YtnP primarily disrupts the AbaI/AbaR QS regulatory cascade, thereby downregulating biofilm-associated virulence genes. It does not exclude the potential involvement of other endogenous regulatory pathways such as *BfmRS* in modulating *ompA* and *csuA/B* expression without imposing classical bactericidal pressure.

### Enzymatic behavior and catalytic ambiguity

YtnP exhibits an atypical metal responsiveness distinct from canonical AHL lactonases. It maintains full catalytic activity without exogenous Zn^2+^ supplementation, whereas its activity is markedly suppressed by EDTA and excess exogenous Zn^2+^. This phenomenon indicates that YtnP possesses a structurally rigid and conformationally constrained metallohydrolase pocket with pre-occupied catalytic metal coordination; excessive external Zn^2+^ disrupts the precise geometric arrangement of the active site rather than further enhancing catalysis. Although the conserved HXHXDH motif is predicted to mediate metal coordination (putatively Zn^2+^ or Fe^2+^), the exact endogenous metal cofactor and atomic coordination mode remain to be verified by ICP-MS and high-resolution structural crystallography (Ušjak et al., 2023). We hypothesize that the HXHXDH motif accommodates a specific metal ion (likely Zn^2+^ or Fe^2+^) in a precise coordination geometry, which is disrupted by competing cations or metal overload.

Host serum and epithelial fluids maintain micromolar free Zn^2+^; enzymes reliant on labile metal binding often suffer from misfolding or substrate competition *in vivo*. By contrast, YtnP’s insensitivity to exogenous metal fluctuations likely represents an evolutionary adaptation to the host microenvironment, ensuring functional stability in complex biological matrices. This distinction implies that YtnP is a structurally MBL-fold hydrolase with a catalytically specialized mechanism, warranting future validation via structural biology and site-directed mutagenesis (e.g., H113A, D115A, H191A).

### Biofilm disassembly and transcriptional impact

Functionally, the most clinically relevant outcome is the disruption of established 24 h CRAB biofilms, a stage notoriously tolerant to antibiotics and immune effectors (Yadav et al., 2024). Beyond bulk AHL removal, YtnP treatment significantly downregulated *ompA* and *csuA/B* expression, coinciding with a COMSTAT-level collapse of biomass and maximum thickness. We interpret this not as evidence that *ompA* or *csuA/B* are exclusive slaves of 3-OH-C12-HSL–both operons are also regulated by *BfmRS* and environmental cues–but as a composite effect of direct QS suppression and feed-forward structural collapse, vividly captured by the shift from SYTO-9– to propidium iodide–dominant architectures.

### Non-bactericidal mechanism underpinning anti-virulence activity

Critically, this biofilm-disrupting and virulence-attenuating activity occurs independently of direct bactericidal effects. Planktonic growth assays confirmed that YtnP (50 μg/mL, 2.5 U/mL) does not inhibit CRAB viability: no statistically significant differences in OD_600_ (12 h kinetic monitoring) or CFU counts (0, 6, 12 h enumeration) were observed between YtnP-treated and PBS-control groups (*p* > 0.05). This lack of bactericidal activity aligns with the core principle of anti-virulence therapy–disarming pathogenicity without imposing selective pressure for resistance–and distinguishes YtnP from conventional antibiotics that target essential bacterial processes (e.g., cell wall synthesis or protein translation). By sparing planktonic growth, YtnP avoids the evolutionary arms race driven by bactericidal agents, making it a sustainable adjuvant for combating CRAB’s multidrug resistance.

We acknowledge notable differences in local 3-OH-C12-HSL concentration and microenvironmental conditions between planktonic and biofilm-embedded CRAB cells. Biofilm matrices trap secreted QS signals, creating high local autoinducer levels that drive robust virulence and biofilm gene expression; by contrast, planktonic cultures contain dilute AHL concentrations with weaker QS activation. Our planktonic growth assays serve as critical baseline controls to exclude universal cytotoxicity of YtnP: if the enzyme inhibited or killed CRAB, such effects would manifest in both physiological states. The unchanged viability of planktonic cells confirms that the drastic loss of biofilm structural integrity and suppressed biofilm gene transcription (*ompA*, *csuA/B*) observed in biofilm populations stem specifically from QS signal hydrolysis, rather than direct bacterial killing. Notably, all qRT-PCR assays measuring biofilm-associated gene expression were performed on RNA extracted from intact biofilm aggregates, directly capturing transcriptional responses of matrix-resident CRAB cells as suggested by the reviewer.

### YtnP attenuates CRAB supernatant-mediated toxicity in a *G. mellonella* model

To isolate the contribution of diffusible QS-dependent virulence factors from direct bacterial killing, we employed a cell-free CRAB supernatant model in *G. mellonella* (Pereira et al., 2020). This experimental design decoupled the contribution of diffusible virulence factors from direct bacterial killing, allowing us to isolate the specific impact of QS disruption. Inoculation with CRAB metabolites alone induced rapid larval lethality, reducing survival to 10.00% within 36 h. Crucially, co-administration of active YtnP conferred a robust protective effect, elevating 36-h survival to 63.33%–a nearly sixfold increase over the untreated CRAB control. This protection was strictly dependent on enzymatic activity, as heat-inactivated YtnP failed to improve survival, thereby excluding nonspecific protein effects or physical sequestration of toxic molecules. These findings demonstrate that YtnP effectively disarms the QS-driven virulence circuitry of CRAB in a physiologically relevant host environment, mitigating metabolic toxicity without imposing bactericidal pressure.

While these results underscore the therapeutic promise of YtnP, the *G. mellonella* model has inherent limitations. The *Galleria mellonella* model lacks adaptive immunity and mammalian tissue microenvironment complexity. Accordingly, the current *in vivo* data only reflect the protective effect of YtnP against QS-related diffusible virulence factors, and cannot fully recapitulate host–pathogen interactions during live CRAB infection in mammals. Definitive validation will therefore require mammalian infection models, such as murine pneumonia or catheter-associated infection, ideally incorporating *abaI* knockout strains and exogenous AHL rescue experiments to confirm on-target specificity. From a translational perspective, YtnP is best framed not as a standalone systemic drug but as a biofilm-disrupting adjuvant (Curcic et al., 2024).

Although non-bactericidal QQ intervention theoretically generates weaker selection pressure than lethal antibiotics, CRAB could evolve multiple adaptive strategies upon sustained exposure to lactonase. As reported in existing quorum-sensing research, adaptive mutants may arise via mutations in the AbaI AHL synthase or AbaR receptor to restore signal communication, overexpression of efflux transporters to eliminate extracellular QQ enzymes, or activation of QS-independent virulence regulatory pathways to bypass the AbaI/AbaR circuit. Since the current study only performed short-term *in vitro* and short-term *in vivo* assays without serial long-term passage experiments to screen QQ-resistant CRAB variants, we cannot definitively conclude that YtnP effectively suppresses resistance formation. Further serial evolution culture experiments are needed to systematically characterize potential adaptive mutations triggered by persistent YtnP treatment.

### Limitations and future directions

This study has several inherent limitations that should be acknowledged. First, all mechanistic experiments were performed on a single clinical CRAB isolate; further validation with multiple independent clinical strains is required to generalize our findings. Second, although molecular docking and conserved motif analysis were performed, the precise catalytic mechanism and key amino acid residues of YtnP need further verification via site-directed mutagenesis and high-resolution structural analysis. Third, only cell-free supernatant was used in the *Galleria mellonella* model; live bacterial infection and mammalian infection models are still needed to better evaluate its *in vivo* protective effect. Fourth, the reduction in biofilm biomass may be partially caused by mechanical detachment of loosely adhered cells during sample processing; future *in situ* non-wash detection is required to distinguish structural collapse from actual cell death. Fifth, pH stability of YtnP was only assessed at 25 °C rather than physiologically relevant 37 °C and storage-relevant 4 °C. Multi-temperature pH stability screening will be carried out in our follow-up applied research. Sixth, long-term serial passage experiments to evaluate the emergence of CRAB adaptive resistance under continuous YtnP stress were not conducted in this study. So future work will perform continuous subculture screening to identify potential resistant mutants and their molecular mechanisms. Finally, the synergistic effects of YtnP with tigecycline or colistin remain uncharacterized by standardized checkerboard assays. Addressing these limitations will further support the translational application of YtnP as a promising anti-virulence adjuvant.

In addition, the heat-inactivated YtnP control used in the *Galleria mellonella* assay was only verified for complete loss of catalytic activity via HPLC; we did not conduct circular dichroism, size-exclusion chromatography or native PAGE to characterize protein conformational changes after 95 °C treatment. Future related research will adopt mild thermal inactivation conditions and supplementary structural detection to further optimize the control design.

In future research, we will expand the range of target virulence genes detected by qRT-PCR to further dissect the regulatory network mediated by YtnP in CRAB biofilms.

In summary, while YtnP-mediated degradation of *A. baumannii* 3-OH-C12-HSL has been previously documented, this work provides comprehensive mechanistic and functional evidence that mangrove rhizosphere-derived YtnP from *B. licheniformis* T2 effectively disarms clinical CRAB via hydrolyzing its master QS signal, downregulating virulence-related outer membrane proteins, and protecting eukaryotic hosts from CRAB virulence-induced lethality. Our work highlights the translational potential of mangrove-derived YtnP as a sustainable anti-virulence adjuvant against multidrug-resistant CRAB infections.

## Conclusion

In conclusion, this study fully characterized a thermostable YtnP lactonase isolated from mangrove rhizosphere *Bacillus licheniformis* T2. Consistent with earlier reports showing that YtnP-family enzymes degrade the QS signal 3-OH-C12-HSL of *A. baumannii*, our data further confirm that this mangrove-derived YtnP effectively quenches QS signaling in clinical CRAB, disrupts mature biofilms, and significantly downregulates virulence-related outer membrane protein genes *ompA* and *csuA/B*. Notably, YtnP exerts anti-virulence and antibiofilm effects without direct bactericidal activity.

In theory, such non-lethal intervention imposes weaker selective pressure on CRAB than conventional bactericidal antibiotics. However, CRAB may develop adaptive mutations under prolonged exposure to quorum-quenching enzymes, and long-term serial culture assays are required to determine whether YtnP can decrease the probability of resistance emergence. Our findings indicate that mangrove-derived YtnP serves as a promising adjuvant candidate for treating intractable CRAB infections, and further assessments in mammalian models and combined antibiotic regimens are merited.

## Data Availability

The data presented in the study are deposited in the GenBank repository, accession number PZ805029 and PZ745974.
